# 体外诱导建立NCI-H2228/Crizotinib耐药细胞株的方法学探讨及鉴定分析

**DOI:** 10.3779/j.issn.1009-3419.2015.06.02

**Published:** 2015-06-20

**Authors:** 荻 吴, 贵花 金, 大玮 赵, 越 张, 静 赵, 鸿 于

**Affiliations:** 1 130012 长春，吉林大学第一医院肿瘤中心 Tumor Center, No.1 Hospital of Jilin University, Changchun 130012, China; 2 130024 长春，东北师范大学生命科学学院 School of Life Sciences, Northeast Normal University, Changchun 130024, China; 3 130012 长春，吉林省肿瘤医院乳腺科 Department of Breast Tumor, Jilin Province Tumor Hospital, Changchun 130012, China; 4 130062 长春，吉林大学人兽共患病研究所，人兽共患病研究教育部重点实验室 Key Laboratory for Zoonosis Research, Ministry of Education, Institute of Zoonosis, Jilin University, Changchun 130062, China; 5 130012 长春，吉林省肿瘤防治研究所细胞生物研究室 Cell Biology Laboratory, Jilin Province Tumor Institute, Changchun 130012, China

**Keywords:** 肺肿瘤, 小分子靶向药物, Crizotinib, 耐药, *EML4-ALK*, Lung neoplasms, Small-molecule-targeted drugs, Crizotinib, Drug resistance, *EML4-ALK*

## Abstract

**背景与目的:**

小分子靶向药物发生耐药的机制及寻找克服耐药的手段是目前提高临床疗效需要迫切解决的问题。本研究探讨采用不同方法建立对Crizotinib耐药的非小细胞肺癌NCI-H2228/Crizotinib细胞株的可行性及鉴定分析，为深入研究Crizotinib耐药发生的机制并寻找克服耐药的手段提供实验基础和理论依据。

**方法:**

采用逐步增加药物浓度和化学诱变剂处理NCI-H2228细胞，诱导细胞对Crizotinib耐药。MTT法检测亲本细胞和耐药细胞的50%抑制浓度（50% inhibitory concentration, IC_50_）和群体倍增时间。RT-PCR和Western blot实验检测棘皮动物微管相关蛋白样4-间变性淋巴瘤激酶（echinoderm microtubule-associated protein like 4-anaplastic lymph kinase, EML4-ALK）基因表达。对耐药细胞和亲本细胞的*EML4-ALK*基因全长测序并对比分析发生耐药的机制。

**结果:**

逐步增加药物浓度的方法耗时过长，细胞恢复生长缓慢，不能有效诱导NCI-H2228细胞对Crizotinib耐药；化学诱变剂ENU可以在短时间内诱导NCI-H2228细胞对Crizotinib耐药[IC_50_=（3.810±1.100）μmol/L，*P*=0.002, 9，*vs*亲本细胞]。耐药细胞*EML4-ALK*基因发生点突变的频率高于亲本细胞。

**结论:**

化学诱变剂诱导细胞耐药操作简便，可有效缩短实验流程，为深入研究耐药发生机制，寻找克服靶向药物耐药的手段提供了前期技术方法和实验依据。

## Abstract (en)

**Background and objective:**

The mechanisms of small molecule targeting drug resistance and ways to overcome resistance are now both urgent need to improve the clinical efficacy. This study aimed to investigate the feasibility of using different methods to establish the crizotinib-resistant non-small cell lung cancer NCI-H2228/Crizotinib cell lines and to clarify the mechanisms of resistance to small molecule targeting drug, thus providing experimental and theoretical bases for further studies to overcome the mechanisms of Crizotinib resistance.

**Methods:**

The study utilized stepwise increase of drug concentrations and chemical mutagen to induce Crizotinib-resistant NCI-H2228 cells. The drug 50% inhibitory concentration (IC_50_) values of parental and resistant cells and the population doubling time were determined by MTT assay. The echinoderm microtubule-associated protein-like 4-anaplastic lymphoma kinase (EML4-ALK) expression was evaluated by RT-PCR and Western blot. Full-length sequencing method was used to compare the *EML4-ALK* genes in the parent and drug-resistant cells and analyze the mechanisms of drug resistance.

**Results:**

The method of gradually increasing drug concentration to induce Crizotinib-resistant NCI-H2228 cells was time-consuming because the cell growth recovery was extremely slow. Thus, this method was considered invalid. However, chemical mutagen ENU can effectively induce NCI-H2228 cells resistant to crizotinib in a short time [IC_50_]= (3.810±1.100) μmol/L, *P*=0.002, 9 *vs* parental cells]. Furthermore, the gene mutation frequency of *EML4-ALK* in the resistant cells was significantly higher than that in the parent cells.

**Conclusion:**

Chemical mutagen-induced cell resistance was easily operated and had effectively shortened the experimental process. Preliminary technical methods and experimental evidence for in-depth study of drug resistance mechanisms and approaches to overcome the targeted drug resistance were also provided.

肺癌是目前发病率和病死率较高的肿瘤之一，其中非小细胞肺癌（non-small cell lung cancer, NSCLC）占肺癌总数的80%-85%。晚期NSCLC的治疗以放、化疗为基础的综合治疗为主。近年来，随着分子生物学研究的发展，靶向治疗逐渐用于临床治疗并获得了良好的疗效。自从2007年首次在NSCLC组织中发现棘皮动物微管相关蛋白样4-间变性淋巴瘤激酶（echinoderm microtubule-associated protein like 4-anaplastic lymphoma kinase, *EML4-ALK*）基因融合现象以来^[1]^，*EML4-ALK*融合基因已经成为NSCLC分子分型的生物标志物和个体化治疗的新靶点。小分子靶向药物Crizotinib是一个MET/ALK/ROS1小分子酪氨酸激酶抑制剂（tyrosine kinase inhibitor, TKI），已于2011年被美国食品药品监督管理局（Food and Drug Administration, FDA）批准用于治疗ALK阳性的NSCLC。虽然Crizotinib疗效显著，但是通常在1年内也都会不可避免的发生耐药^[2]^。由于NSCLC中ALK融合基因本身具有的结构上的复杂性，Crizotinib耐药机制相比其他的靶向药物也更加复杂，涉及基因突变、基因拷贝数增益、信号转导通路等，其确切机制至今尚未被完全阐明^[3, 4]^。

发生在ALK激酶域（kinase domain, KD）的点突变是产生耐药的主要机制，这些突变主要通过产生空间位阻妨碍Crizotinib与其靶点的结合^[5]^。已有的报道^[6]^表明，临床发生Crizotinib耐药的患者涉及到点突变差异较大。为深入研究耐药发生的机制，我们设想在体外建立一个对Crizotinib获得性耐药的细胞模型，通过增加药物浓度和/或快速诱变处理一个表达EML4-ALK的NSCLC细胞系NCI-H2228，然后比较耐药细胞与亲本细胞的IC_50_差异并分析*EML4-ALK*基因的改变，为深入分析耐药机制和寻找克服耐药的手段提供前期的技术方法和实验依据。

## 1 材料和方法

### 1.1 材料

RPMI-1640购自Hyclone，IMDM购自Gibco；胎牛血清购自BioInd（FOETAL BOVINE SERUM，Cat.04-001-1A）；人NSCLC细胞株NCI-H2228购自ATCC（NCI-H2228, Human Lung Adenocarcinoma, Lot: 59868851）；NSCLC A549细胞株由东北师范大学生命科学学院提供；Crizotinib购自Selleck Chemicals（Cat.S1068）；紫杉醇（6 mg/mL）注射液购自山东普德药业股份有限公司。Nethyl-N-nitrosourea（ENU）购自Sigma Aldrich；RNeasy Mini Kit（Cat. 74126）、QuantiTect reverse transcription kit（Cat.205313)均为Qiagen公司产品；PCR引物由上海捷瑞生物工程有限公司合成。ALK（C26G7) rabbit mAb（Cat.3333）和phospho-ALK（Y1604）antibody（Cat.3341）购自Cell Signaling Technology。

### 1.2 细胞培养

NCI-H2228细胞培养在RPMI-1640培养基中（含10%FCS、100 U/mL青霉素和100 μg/mL链霉素），此细胞株表达*EML4-ALK*融合基因的variant 3（Crizotinib的作用靶点）。A549细胞常规培养于IMDM培养基中。0.25%胰蛋白酶+0.02% EDTA（1:1）消化传代。Crizotinib溶于DMSO，浓度为50 mmol/L，-80 ℃保存备用。ENU溶于DMSO，使用终浓度100 mg/mL。

### 1.3 药物浓度递增法诱导Crizotinib耐药细胞株

采用逐步递增Crizotinib浓度和大剂量冲击交叉作用的方法诱导H2228细胞对Crizotinib耐药。药物起始浓度50 nmol/L，作用72 h后，加入新鲜培养基继续培养，待细胞恢复正常生长后，再用50 nmol/L Crizotinib作用72 h，如此反复两次后，半对数增加Crizotinib浓度。在诱导耐药的过程中，可视细胞生长情况调整加药浓度或加入SCF（100 ng/mL）促进细胞生长。

### 1.4 ENU处理诱导Crizotinib耐药细胞株^[7, 8]^

H2228细胞生长至指数生长期时（5×10^5^个/mL）加入ENU（100 μg/mL），24 h后细胞用1640洗3次，继续培养48 h并加入不同浓度的Crizotinib处理细胞（0、400 nmol/L、800 nmol/L、1, 200 nmol/L、1, 600 nmol/L），视细胞生长情况更换新鲜培养基或持续加药直至细胞恢复生长，最终维持药物耐受浓度为1, 200 nmol/L。

### 1.5 测定IC_50_值

文献^[6, 9]^报道A549细胞是一个NSCLC细胞系，不表达*ALK*融合基因，但是表达野生型EGFR并含有*K-Ras*突变，本实验采用A549细胞作为对照细胞，以比较靶向药物Crizotinib对不同NSCLC细胞的杀伤作用。实验采用Paclitaxel作为对照药物。取对数生长期H2228亲本细胞和耐药细胞及A549细胞，消化后调整细胞浓度为4×10^4^个细胞/mL接种96孔板，100 μL/孔，24 h后，加入不同浓度的实验药物（Crizotinib起始浓度100 μmol/L；Paclitaxel起始浓度7.034 μmol/L，依次10倍稀释共6个-7个浓度），实验同时设空白组、培养液对照组和溶剂对照组，每组4个平行孔，37 ℃、5%CO_2_、饱和湿度条件下培养72 h，MTT法检测细胞在490 nm处的吸光度值，SPSS 17.0软件计算IC_50_值。

### 1.6 H2228亲本细胞和耐药细胞的群体倍增时间的测定计算

细胞消化后制成单细胞悬液并调整细胞浓度为1×10^5^个细胞/mL，按1 mL/孔将细胞接种于24孔培养板。从第2天开始每天计数细胞共7天。以培养时间（天数）为横轴，细胞数为纵轴，绘制细胞生长曲线。根据公式：Td(h)=t(h)×[log2/(logNt-logNo)]，计算细胞群体倍增时间。其中Td为群体倍增时间，t为对数增殖期时间，Nt为对数增殖期结束时的细胞数，No为对数增殖期开始时的细胞数。

### 1.7 细胞总RNA提取和RT-PCR

用RNeasy Mini Kit提取细胞总RNA，按照试剂盒说明书操作。取1 μg总RNA用于cDNA合成，采用QuantiTect reverse transcription kit，按照试剂盒说明书操作。PCR引物见表 1、表 2。ALK（Genbank U62540）引物V对应ALK的胞外域（可用于检测野生型ALK mRNA），引物Ⅲ对应ALK的激酶域。引物EML4-F和EML4-R跨越EML4的3′端。*GAPDH*基因作为内参照。耐药细胞和亲本细胞*EML4-ALK*基因测序由上海生工完成。测序产物经拼接后做blast比对分析。

**1:** PCR引物 PCR primer

Name	Sequence	Product (bp)
GAPDH-F	5′-GTCAGTGGTGGACCTGACCT-3′	212
GAPDH-R	5′-TGAGCTTGACAAAGTGGTCG-3′	
E6A20-F	5′-TTCGAGCATCACCTTCTCC-3′	515
E6A20-R	5′-GGACACCTGGCCTTCATAC-3′	
EML4-F	5′-CAGCCAT-GTCACCAATGTC-3′	302
EML4-R	5′-CACTTGG-CTCCACAGTTTGT-3′	
ALK-V-F	5′-ATGGTGTTGCCTCTCCTCGATGTGTC-3′	693
ALK-V-R	5′-CGTAGGTGGCTCCACCCCCTCC-3′	
ALK-Ⅲ-F	5′-GGGCCATGGCGCCTTTGGGGAGGT-3′	760
ALK-Ⅲ-R	5′-GTTGGGCCTGTCTTCAGGCTGATGTTGC-3′	
1-F	5′-AAGTGCCCGCCCCTCTAA-3′	773
1-R	5′-GCAGCTCCTGGTGCTTCC-3′	
2-F	5′-CATCATCAACCAAGCAAA-3′	596
2-R	5′-CCAAATACTGACAGCCACA-3′	
3-F	5′-CCCCGGTTCATCCTGCTG-3′	683
3-R	5′-GGGTCCTTGGGCCTCACA-3′	
4-F	5′-CAACACCGCTTTGCCGATA-3′	697
4-R	5′-GTGCGACCGAGCTCAGGG-3′	

**2:** PCR反应条件 PCR reaction conditions

Fragment	Reaction conditions (Taq polymerase)
GAPDH	94℃ 5 min; 94℃ 30 s, 55℃ 30 s, 72℃ 30 s, 25 cycles; 72℃ 5 min
EML4	95℃ 5 min; 95℃ 30 s, 58℃ 30 s, 72℃ 1 min, 40 cycles; 72℃ 10 min
ALK-Ⅲ	98℃ 5 min; 95℃ 15 s, 60℃ 30 s, 72℃ 30 s, 30 cycles; 72℃ 5 min
E6A20	95℃ 5 min; 95℃ 30 s, 54℃ 30 s, 72℃ 2 min30 s, 35 cycles; 72℃ 10 min
ALK-Ⅴ	98℃ 1 min; 95℃ 15 s, 60℃ 30 s, 72℃ 30 s, 30 cycles; 72℃ 5 min
Prime 1, 3	94℃ 5 min; 94℃ 15 s, 52.5℃ 30 s, 72℃ 30 s, 35 cycles; 72℃ 5 min
Prime 2, 4	94℃ 5 min; 94℃ 30 s, 54℃ 30 s, 72℃ 30 s, 35 cycles; 72℃ 5 min

### 1.8 Western blot检测ALK和P-ALK蛋白表达

A549和H2228细胞计数1×10^6^个细胞，用100 μL lysis buffer裂解细胞（10 mM sodium fluoride, 1 mM sodium orthovanadate, 1 mM leupeptin, 1 mM pepstatin, 1 mM aprotinin, 20 mM PMSF）。离心后取上清用Bradford' s实验确定蛋白含量（含50 μg-100 μg蛋白），7.5% SDS-PAGE凝胶电泳，蛋白转移到PVDF膜上。膜封闭在5% BSA中，室温45 min，用TBST洗膜3次，每次10 min。一抗（1:1, 000）孵育，室温1 h，二抗（1:2, 000）孵育，室温45 min，化学发光法检测特异性条带^[10]^。

### 1.9 统计学方法

应用SPSS 17.0统计学软件进行分析，计量数据采用Mean±SD表示，使用*t*检验或方差分析。以*P* < 0.05为差异有统计学意义。

## 2 结果

### 2.1 耐药细胞形态学观察和IC_50_测定

H2228细胞呈不规则的多角形，细胞生长的速度低于其他的肺癌细胞（如A549细胞），而且细胞贴壁作用强，难于消化，培养过程中，细胞约7 d-10 d传代一次。我们采用增加药物浓度的方法诱导耐药细胞，发现经Crizotinib处理后，H2228的细胞形态发生了改变，细胞在加药后变得比亲本细胞大，部分细胞变圆，开始悬浮生长，继而死亡，贴壁细胞的形态变得更加不规则（图 1）。由于H2228细胞的生长速度过于缓慢，加药处理后细胞恢复生长所需的时间过长（Crizotinib浓度 < 500 nmol/L，细胞培养4个月左右，虽然细胞状态趋于正常，但存活细胞没有扩增），因此诱导后细胞很难恢复生长到足够数量用于后续实验。经过长期反复实验，我们认为这种方法可行性较差，所以我们尝试用化学诱变剂处理H2228细胞诱导细胞对Crizotinib耐药。N-ethyl-N-nitrosourea（ENU）是一种强烈的点突变诱变剂，可随机诱导多个蛋白突变。本研究结果表明ENU处理细胞后，虽然漂浮的细胞数量增多，但细胞形态并没有发生明显的改变而且生长速度恢复较快，诱导后耐药细胞对Crizotinib的IC_50_值（3.810±1.100）μmol/L明显高于亲本细胞（1.003±0.372）μmol/L（*P*=0.0029, *n*=4）。Crizotinib对A549细胞的IC_50_值为（3.345±1.625）μmol/L，与耐药细胞的IC_50_值接近，这一结果与Katayama等^[11]^的报道一致，他们采用逐步增加Crizotinib浓度的方法建立了H3122 CR（表达EML4-ALK variant 1）耐药细胞株，其IC_50_值与不表达*ALK*基因重排的A549等肺癌细胞的IC_50_值均大于1 μmol/L。

**1:**
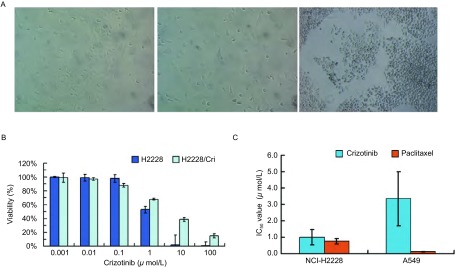
Crizotinib诱导细胞耐药形态学观察和IC_50_值的比较。A：NCI-H2228（左）和NCI-H2228/Crizotinib（中：药物浓度递增组；右：ENU诱变组；×100）的细胞形态（培养起始浓度均为5×10^5^ cells/mL）；B：Crizotinib处理后NCI-H2228和NCI-H2228/Crizotinib的细胞存活率（%）；C：NCI-H2228和A549细胞IC_50_值的比较。 Cell morphology after Crizotinib induced drug resistant and the results of IC_50_ values. A: Cell morphology of NCI-H2228 (left) and NCI-H2228/ Crizotinib (Middle: increasing drug concentration group; Right: ENU mutagenesis group) (×100); Note: The initial cell concentration was 5×10^5^ cells/mL in each group. B: Cell viability (%) of NCI-H2228 and NCI-H2228/Crizotinib after Crizotinib treatment; C: Comparison of IC_50_ values of NCI-H2228 and A549 cells. IC_50_: 50% inhibitory concentration.

### 2.2 亲本细胞和耐药细胞的群体倍增时间的比较

将亲本细胞和耐药细胞制成单细胞悬液，以相同浓度分别接种于24孔板，连续7天计数细胞，绘制细胞生长曲线，按照公式计算细胞群体倍增时间。实验结果表明（图 2），ENU处理诱导的H2228/Crizotinib耐药细胞的生长速度较亲本细胞降低，其群体倍增时间为（313.40±50.65）h[亲本细胞的群体倍增时间为（250.80±58.29）h，*P*=0.028, 1，*n*=3]。

**2:**
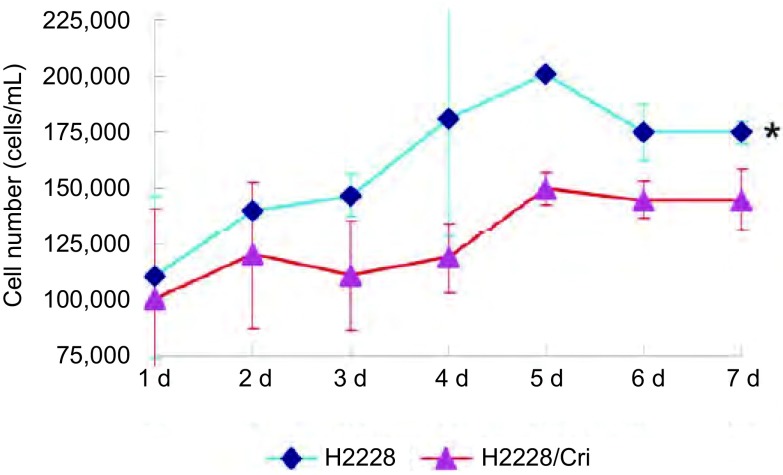
H2228亲本细胞和耐药细胞的生长曲线。*P < 0.05, H2228/Cri *vs* H2228。 Growth curve of H2228 parental cells and resistant cells.*P < 0.05, H2228/Cri *vs* H2228.

### 2.3 PCR实验结果

实验首先比较了H2228和A549细胞EML4-ALK表达情况，结果表明H2228细胞表达EML4、ALK Ⅲ和EML4-ALK（variant 3b），A549细胞表达EML4，不表达EML4-ALK（variant 3b），与文献^[8]^报道一致。细胞经ENU处理诱导产生的H2228/Crizotinib耐药细胞在相应引物所涉及的基因区域没有发生明显的改变（仅对应ALK激酶域的条带显示比对照细胞低表达），分析原因可能是诱导的突变并没有发生在这些部分，或诱导发生的基因突变没有改变DNA的二级结构，不足以体现出结果上的差异。引物ALK V的PCR扩增结果显示没有在693 bp处出现条带，进一步说明该细胞并不是ALK野生型的细胞（图 3、表 3）。

**3:**
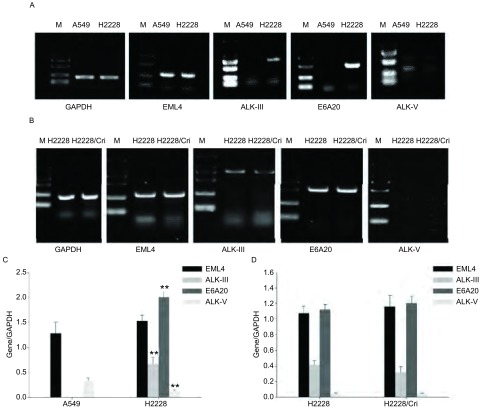
Crizotinib耐药前后H2228细胞中*EML4-ALK*融合基因表达的变化。A：A549和H2228细胞；B：H2228和H2228/Crizotinib细胞；C：对图A结果的统计分析；D：对图B结果的统计分析。**P < 0.01, A549 *vs* H2228。 The expression of EML4-ALK in H2228 cells before and after Crizotinib resistant induction. A: A549 cells and H2228 cells; B: H2228 cells and H2228/Crizotinib cells. C: Statistical analysis of the results of A; D: Statistical analysis of the results of B. **P < 0.01, A549 *vs* H2228. EML4-ALK: echinodermmicrotubule-associated protein like 4-anaplastic lymphoma kinase.

**3:** PCR实验结果分析 Analysis of the PCR assay results

Fragment	*P* value	
	A549 *vs* H2228	H2228/Cri *vs* H2228
EML4	0.175	0.422
ALK-Ⅲ	0.001	0.156
E6A20	0.001	0.284
ALK-V	0.004	0.312

### 2.4 Western blot实验结果

上述PCR实验结果表明亲本细胞表达EML4-ALK variant 3b（AB374362; BAG55004），蛋白质长度为796 aa，分子量87.635 kDa。本实验采用ALK和P-ALK抗体检测了H2228亲本细胞和耐药细胞相应蛋白的表达。结果表明对照细胞A549、H2228亲本细胞和耐药细胞均表达ALK和P-ALK[*P*=0.007 (ALK), A549 *vs* H2228; *P*=0.529 (P-ALK), A549 *vs* H2228]，同时H2228/Crizotinib耐药细胞ALK蛋白的表达与亲本细胞相比均有明显差异[*P*=0.002 (ALK)，H2228/Cri *vs* H2228；*P*=0.001 (P-ALK)，H2228/Cri *vs* H2228，图 4]，说明ENU处理可能通过改变ALK和P-ALK蛋白表达诱导H2228细胞耐药。

**4:**
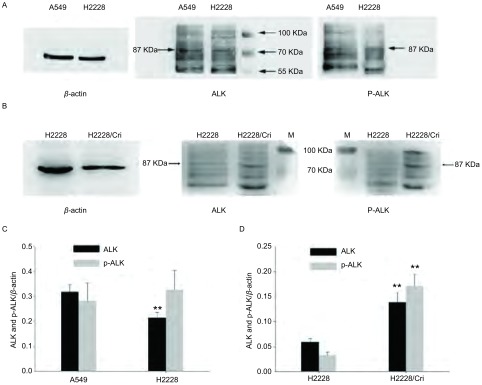
A549、H2228和H2228/Crizotinib细胞ALK和P-ALK蛋白表达分析。A：A549和H2228细胞ALK和P-ALK蛋白表达；B：H2228和H2228/Crizotinib细胞ALK和P-ALK蛋白表达；C：对图A结果的统计分析；D：对图B结果的统计分析。**P < 0.01, A549 *vs* H2228; *P* < 0.01, H2228/Cri *vs* H2228。 ALK and P-ALK expression of A549 cells, H2228 cells and H2228/Crizotinib cells. A: ALK and P-ALK expression of A549 and H2228 cells; B: ALK and P-ALK expression of H2228 and H2228/Crizotinib cells. C: Statistical analysis of the results of A; D: Statistical analysis of the results of B. **P < 0.01, A549 *vs* H2228; *P* < 0.01, H2228/Cri *vs* H2228.

### 2.5 测序结果的分析

针对人类EML4-ALK variant 3b（GenBank: AB374362.1；全长2, 506 bp）我们设计的PCR引物扩增出4个PCR产物，分别对应*EML4-ALK*基因的相应位置。无Crizotinib处理的对照组的测序结果经拼接后得到了全长的基因序列，经Blast比对，与EML4-ALK variant 3b序列一致。经ENU诱变剂处理的Crizotinib耐药组的测序结果经拼接后得到一条长为2, 417 bp的基因序列，涵盖了EML4-ALK variant 3b的CDS区（68 bp-2, 458 bp），在与对照组比较后，发现全长序列均有点突变的发生。进一步将所获得的测序结果与文献^[5-7]^报道的诱发Crizotinib耐药的*ALK*基因的二次突变（L1196M、C1156Y、F1174L、L1152R、G1269A、G1202R、S1206Y、1151Tins、I1171、F1174C、D1203N等）对比分析后发现没有已知的点突变发生，但是在ALK基因的酪氨酸激酶域和ATP结合位点部分共有4个错配和1个插入突变发生，推测这些突变的发生与细胞发生耐药相关（图 5）。

**5:**
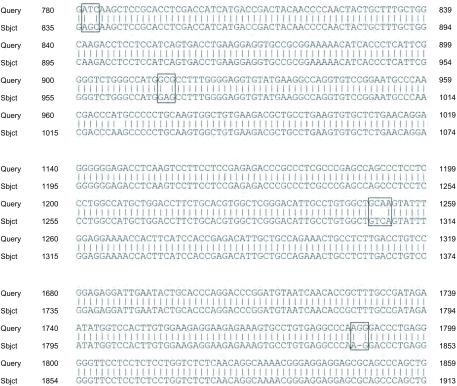
H2228细胞和H2228/Crizotinib细胞测序结果的比对分析 Comparative analysis the sequencing results of H2228 cells and H2228/Crizotinib cells

## 3 讨论

2007年Soda等^[1]^发现一种新的癌基因*EML4-ALK*参与了NSCLC的发生过程。EML4是棘皮动物微管相关蛋白样蛋白质家族成员，其结构组成包含一个N-端基本区，一个疏水的棘皮动物微管相关蛋白样蛋白（hydrophobic echinoderm microtubule-associated protein-like protein, HELP）域和WD重复区。ALK与其他基因（如*NPM*、*EML4*等）的融合已在多种恶性肿瘤中被发现，针对ALK激酶域的靶向药物治疗是目前肺癌治疗领域最受关注的焦点。

*EML4-ALK*融合基因有多个亚型，所有的亚型都含有ALK胞内酪氨酸激酶结构域^[12]^。2008年有研究^[13]^在2例NSCLC患者中发现并鉴定了*EML4-ALK*亚型3a和3b。其中亚型3a是EML4的外显子6与ALK的外显子20融合的产物，亚型3b在3a的融合点之间另含有33 bp的序列，对应EML4的内含子6。EML4-ALK亚型3a和3b都显示出不低于亚型1和2的转化活性和激酶活性。2009年Wong等^[14]^报道了中国NSCLC患者EML4-ALK的融合频率、基因表达谱和临床病理特征。在266例手术切除的原发NSCLC标本中13例（4.9%）表达EML4-ALK，其中8例表达variant 3a/b。张绪超等^[15]^报道一组103例NSCLC患者中*EML4-ALK*融合基因的频率，确定12例（11.6%）表达EML4-ALK。其中3例表达variant 3a/b。国外研究者^[16]^在临床检测中也发现含有*EML4-ALK*融合基因的NSCLC中variant 1占比最高，variant 3a/b次之。H2228 NSCLC细胞表达variant 3a/b（E6a/b; A20）EML4-ALK融合蛋白，对ALK激酶抑制剂Crizotinib治疗敏感，因此本研究选择H2228细胞作为研究对象，以克服临床样本来源受限所造成的困难。

临床治疗中尽管ALK TKI Crizotinib对携带*ALK*融合基因的NSCLC患者有显著疗效，通常在1年左右也都会发生对Crizotinib的耐药^[17]^。迄今的研究^[18]^表明发生ALK TKI获得性耐药的机制主要包括激酶域ATP结合位点发生二次突变、EML4-ALK基因拷贝数增益和细胞信号转导旁路的激活等。目前临床治疗发生Crizotinib耐药的患者主要采用使用新的ALK抑制剂、联合用药、再次给药等方式^[11, 19, 20]^，不过实践^[21]^证明不同的*ALK*融合基因和EML4-ALK亚型对Crizotinib的敏感性不同，其机制尚未被阐明。因此为深入研究耐药发生的机制，我们设想建立了一个Crizotinib获得性耐药的细胞模型，研究耐药机制并为克服耐药寻找有效的方法。实验结果表明，采用逐步增加药物浓度的方法诱导NCI-H2228细胞株对Crizotinib耐药虽然是合理的选择，但是H2228细胞本身的生长特点使诱导时间过长，而且产生耐药克隆的产率极低，细胞很难恢复生长到足够数量用于后续实验，实验方法可行性差。我们的实验结果与von Bubnoff等^[22]^的报道相符，他们采用递增药物浓度的方法诱导建立对ABL激酶抑制剂耐药的Ba/F3-p185^BCR-ABL^细胞株，10^6^细胞最高才能产生3.9个耐药克隆。为了解决这一问题，Bradeen等^[8]^建立了使用ENU迅速诱变的实验方法研究Imatinib mesylate、Nilotinib和Dasatinib对Ba/F3-p210^BCR-ABL^细胞的耐药突变谱，证明ENU诱导产生的耐药突变与临床治疗中发生的突变具有高度一致性。ENU是一个强烈的点突变诱变剂，可随机诱导多个蛋白突变，理论上ENU诱导的随机突变可能涉及全部基因，但是细胞经ENU诱变后对小分子靶向药物（如Crizotinib）产生的耐药克隆应该绝大部分是具有ALK激酶域突变特征的，已有的文献^[7, 23]^证实了这一理论上的推测。所以在经过尝试后，我们选择ENU处理H2228细胞诱导耐药，获得的实验结果表明ENU处理细胞后，细胞生长速度恢复较快，诱导后耐药细胞对Crizotinib的IC_50_明显高于亲本细胞（*P* < 0.05），收获获得的耐药细胞数量足以支持后续实验的完成，说明化学诱变剂诱导细胞耐药的方法是可行的。

针对人类EML4-ALK variant 3b（GenBank：AB374362.1；全长2, 506 bp）设计的PCR引物扩增出4个PCR产物，分别对应*EML4-ALK*基因的相应位置。经ENU诱变剂处理的Crizotinib耐药组的测序结果经拼接后得到涵盖全长CDS的EML4-ALK variant 3b基因序列，在与对照组比较后，发现全长序列均有点突变的发生。进一步将所获得的测序结果与文献报道的诱发Crizotinib耐药的ALK基因二次突变（L1196M、C1156Y、F1174L、L1152R、G1269A、G1202R、S1206Y、1151Tins、I1171、F1174C、D1203N、V1180L等）对比分析后发现没有已知的点突变发生，但是耐药细胞在*ALK*基因的酪氨酸激酶域和ATP结合位点部分共有4个错配和1个插入突变发生，这应该是化学诱变后细胞发生耐药的主要机制。此外，Western blot实验结果也表明耐药的H2228/Crizotinib细胞ALK和P-ALK表达增强，说明我们诱导建立的H2228/Crizotinib细胞株由于ALK和P-ALK蛋白表达改变而发生耐药。Zhang等^[7]^报道用ENU处理表达EML4-ALK variant 1的Ba/F3细胞后共发生了422个点突变，其中包括临床报道的L1196M、C1156Y、F1174L等。同时随着Crizotinib浓度的升高（最高1, 440 nmol/L），ALK激酶域发生突变的部位和突变数均呈降低趋势，分析原因可能是由于高浓度药物作用后有能力恢复生长的细胞数明显减少造成的。我们建立的耐药细胞株耐药终浓度为1, 200 nmol/L，药物诱发的基因突变频率与文献报道基本一致，没有检测到已知的点突变的原因可能与培养条件及采用的实验方法有关，因此还需要进一步优化培养体系，深入分析我们检测到的次级突变与药物结合的关系以及相关信号转导通路（ALK、ERK、STAT3等）是否发生改变^[24]^，进而阐明耐药机制，为临床克服耐药提供实验依据。

在NSCLC中*ALK*融合基因的发生率较低，并且还分为多种亚型。由于能获取的临床样本有限，事实上至今还有未知亚型尚未被鉴定，未知的二次突变也还需要深入分析，因此实际发生耐药的机制应该更加复杂。我们得到的测序结果在融合基因的EML4部分也存在突变，这个位置的突变会影响到融合基因的催化活性，这个可能也是诱变后细胞发生耐药的原因之一。总之，我们的研究方法可以在一定程度上解决样本来源受限的问题，技术路线可行，所获得的结果对于阐明耐药发生机制，寻找克服耐药的手段具有实际意义。
